# Activated type 17 helper T cells affect tofacitinib treatment outcomes

**DOI:** 10.1038/s41598-025-87076-7

**Published:** 2025-02-19

**Authors:** Yuki Ito, Daisuke Watanabe, Norihiro Okamoto, Haruka Miyazaki, Eri Tokunaga, Yuna Ku, Makoto Ooi, Namiko Hoshi, Michitaka Kohashi, Maki Kanzawa, Yuzo Kodama

**Affiliations:** 1 Division of Gastroenterology, Department of Internal Medicine, Kobe University Hospital, Kobe, Japan; 2 Department of Gastroenterology, Kakogawa Central City Hospital, Kakogawa, Hyogo Japan; 3 Department of Diagnostic Pathology, Kobe University Graduate School of Medicine, Kobe, Hyogo Japan

**Keywords:** Gastrointestinal diseases, Ulcerative colitis

## Abstract

The incidence of ulcerative colitis (UC) is on the rise also in Japan. Simultaneously, therapeutic options, including biologics and Janus kinase (JAK) inhibitors, have significantly expanded over the past decade. Although tofacitinib (TOF), one of JAK inhibitors, is a viable option for patients with moderate to severe UC, there is insufficient data to predict responsiveness of TOF treatment. The present study aimed to determine whether the infiltration of IL-17 A-positive mononuclear cells into the colonic mucosa can predict responsiveness to TOF treatment. Patients with UC who underwent TOF treatment were divided into responder and failure groups. Subsequently, we conducted a comparative analysis to identify differences in the infiltration of IL-17 A-positive cells into the colonic mucosa through immunohistochemical examination of colon biopsy samples. The proportion of IL-17 A positive mononuclear cells in colon biopsy samples was significantly higher in the failure group than among responders (38.2% vs. 21.2%). Consistent with this finding, our re-analysis of RNA sequence datasets available in the Gene Expression Omnibus (GEO) database suggested that TOF exerts a more pronounced influence on Th1 cells compared with IL-17-producing Th17 cells. In summary, an abundance of IL-17 A-positive mononuclear cells in the colonic mucosa has the potential to predict the responsiveness to TOF treatment.

**Supplementary Information:**

The online version contains supplementary material available at 10.1038/s41598-025-87076-7.

## Introduction

Ulcerative colitis (UC) is a chronic inflammatory bowel disease with an unknown etiology. It is generally considered to develop when genetically susceptible patients encounter environmental factors, such as specific bacteria, diet, and/or medical agents. UC can significantly affect patient quality of life (QOL), and is typically characterized by a relapsing and remitting clinical course. Epidemiological evidence suggests that the incidence of UC has steadily increased worldwide, reaching up to 286 UC cases per 100,000 in the United States^1^. Patients with UC exhibit aberrant innate and adaptive immune responses against microbial antigens. An atypical effector T-helper (Th) cell response and an imbalance in regulatory T cells consequently result in the dysregulated production of key cytokines in inflammation. These cytokines include tumor necrosis factor (TNF), interferon (IFN)-gamma (a Th1-related cytokine), interleukin (IL)-5, IL-6, IL-13 (Th2-related cytokines), IL-17, IL-21, and IL-22 (Th17-related cytokines), and have been implicated in the pathophysiology of UC^2^. In clinical settings, precisely defining histological activity is crucial given the growing acknowledgment of histological remission as a therapeutic endpoint in patients with UC. The Geboes score^3^, Nancy Index^4^, and Robarts Histopathology Index^5^ are among the most commonly used scoring systems, and are valuable for stratifying patients according to histological remission and activity^6^.

Because patients hospitalized for acute severe UC are at particularly high risk for life-threatening complications and emergency colectomy, selecting the optimal management strategy is vital. Therapeutic options, including biologics and Janus kinase (JAK) inhibitors, have significantly expanded over the past decade. Therefore, the current strategy for determining the optimal therapy for moderate-to-severe UC is difficult but with limited guidance regarding the comparative efficacy and safety of different treatments, resulting in considerable practice variability.

JAKs, which have been identified as potent therapeutic targets, demonstrate the potential to interfere with > 50 cytokines^7^. JAKs, which constitutively bind to cytokine receptors, such as the IL receptor, transmit signals through “signal transducers and activators of transcription (STATs)” proteins^7^. JAK and STAT proteins form the JAK-STAT pathway and play crucial roles in biological responses, such as immune regulation. To date, four members of the JAK family (JAK1, JAK2, JAK3, and tyrosine kinase [TYK] 2) and seven members of the STAT family (STAT1, 2, 3, 4, 5a, 5b, and 6) have been documented^7^. Tofacitinib (TOF), one of JAK inhibitors, is an oral and small-molecule pan-JAK inhibitor. Clinical guidelines from the American Gastroenterological Association (AGA) suggest that a JAK inhibitor of TOF is more effective than placebo in inducing and maintaining remission in adult outpatients with moderate to severe UC^8^. Regarding the efficacy of TOF, as highlighted in the placebo-controlled long-term study of CP-690 (OCTAVE trial), 550 in patients with ulcerative colitis demonstrated significant effectiveness in the induction and maintenance of clinical remission^7^. In both the OCTAVE Induction 1 and 2 programs, the primary endpoint of clinical remission at week 8 was more frequently achieved in the TOF 10 mg twice per day (BID) group than in the placebo group (18.5% versus [vs.] 8.2%; *p* = 0.007 for Induction 1; 16.6% vs. 3.6%, *p* < 0.001 for Induction 2). Furthermore, clinical remission at week 52 occurred more frequently in both TOF groups (5 mg and 10 mg BID groups) than in the placebo group in the OCTAVE Sustain trial.

While TOF is a viable option for patients with moderate to severe UC, there are insufficient data to predict responsiveness. As such, the present clinical retrospective study aimed to ascertain whether the infiltration of IL-17 A-positive mononuclear cells can predict responsiveness to TOF treatment using histological analysis.

## Materials and methods

### Study design

This retrospective, observational, single-center study included patients with UC who commenced TOF treatment between May 2018 and April 2022. Baseline clinical data and outcomes were retrieved from medical records. This study aimed to assess the impact of IL-17-positive cell infiltration into the colonic mucosa on the responsiveness to TOF treatment. For the analysis, patients who discontinued TOF were designated as the failure group, whereas those who continued TOF were classified as the responder group. A comparative analysis was performed to determine differences in IL-17 A expression profiles in tissues between the two groups through histological analysis of colon biopsy samples collected before treatment.

### Immunohistopathological examination　

Fresh biopsies obtained during colonoscopy were systematically fixed in 10% formalin for 24 to 48 h. For immunohistopathological analysis, the most severely inflamed lesions observed during endoscopy were selected. Paraffin blocks were sectioned and used for immunostaining. Tissues were immunostained with an IL-17 A antibody (ab79056, Abcam, Cambridge, United Kingdom). Digital images of the immunostained slides were captured using a digital optical microscope. To determine the percentage of IL-17 A-positive mononuclear cells in the tissue, three randomly selected regions, each measuring 100 × 100 μm, were extracted from each histological digital images of the patient samples. Subsequently, the total counts of mononuclear cells and IL-17 A-positive mononuclear cells in each extracted area were determined. Finally, average proportions of IL-17 A positive mononuclear cells were calculated. This investigation was performed in collaboration with a certified pathologist (M.K.).

### Gene expression data from a public data source

Publicly accessible gene expression data were obtained from the Gene Expression Omnibus (GEO) database, specifically with accession numbers GSE60482 and GSE169146, and used to examine the influence of TOF on lymphocytes and patients. The identification of differentially expressed genes (DEGs) was performed using the DESeq2 R package. DEGs were identified as genes meeting the defined criteria of *p* < 0.05 and a log fold change of > 1 or < -1.

### Statistical analysis

Comparisons between continuous variables were performed using Student’s t-test for parametric data and the Mann–Whitney U test for non-parametric data. Categorical data were analyzed using Fisher’s exact test or the chi-squared test, as appropriate. Receiver operating characteristic (ROC) curves were generated to assess the areas under the ROC curves (AUC). The cut-off value was determined using the Youden Index, as revealed by the ROC curves. A threshold of *p* < 0.05 defined statistical significance in all analyses.

## Results

### Patient characteristics

**Fig. 1:**
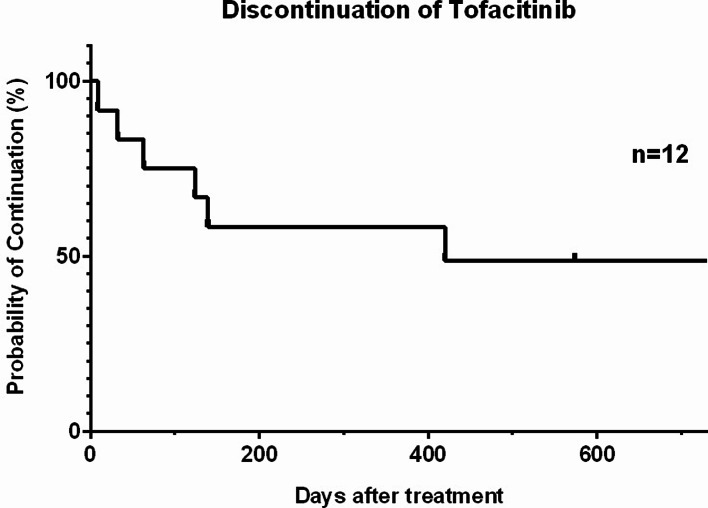
Probability of Tofacitinib (TOF) Continuation. Among the eligible cohort, six individuals discontinued Tofacitinib due to exacerbated disease activity, whereas the remaining patients continued treatment.

Twelve patients with UC, in whom TOF was initially administered between April 2018 and October 2021, were included in this study. Among all patients, six discontinued TOF due to aggravated disease activity, while others continued (Fig. 1 and Supplementary Table 1).

Patients with UC treated with TOF at the authors’ facility are clearly divided into two groups: failure and responder. As such, it was suspected that clinical factors contribute to the response to TOF treatment. Accordingly, biopsy samples were obtained from the most severely inflamed sites of the colorectal mucosa. Three of six patients in the responder group did not undergo colonoscopy before TOF treatment and, therefore, were excluded from further analysis. Finally, nine patients who were eligible for the study were analyzed. Baseline characteristics of the patients are summarized in Table 1. The mean age was 37.4 years, three (33.3%) patients were female, the mean disease duration was 91.4 months, and seven (77.8%) patients had total colitis (Table 1).

### Relationship between the response to TOF and IL-17a-positive immune cells

**Fig. 2:**
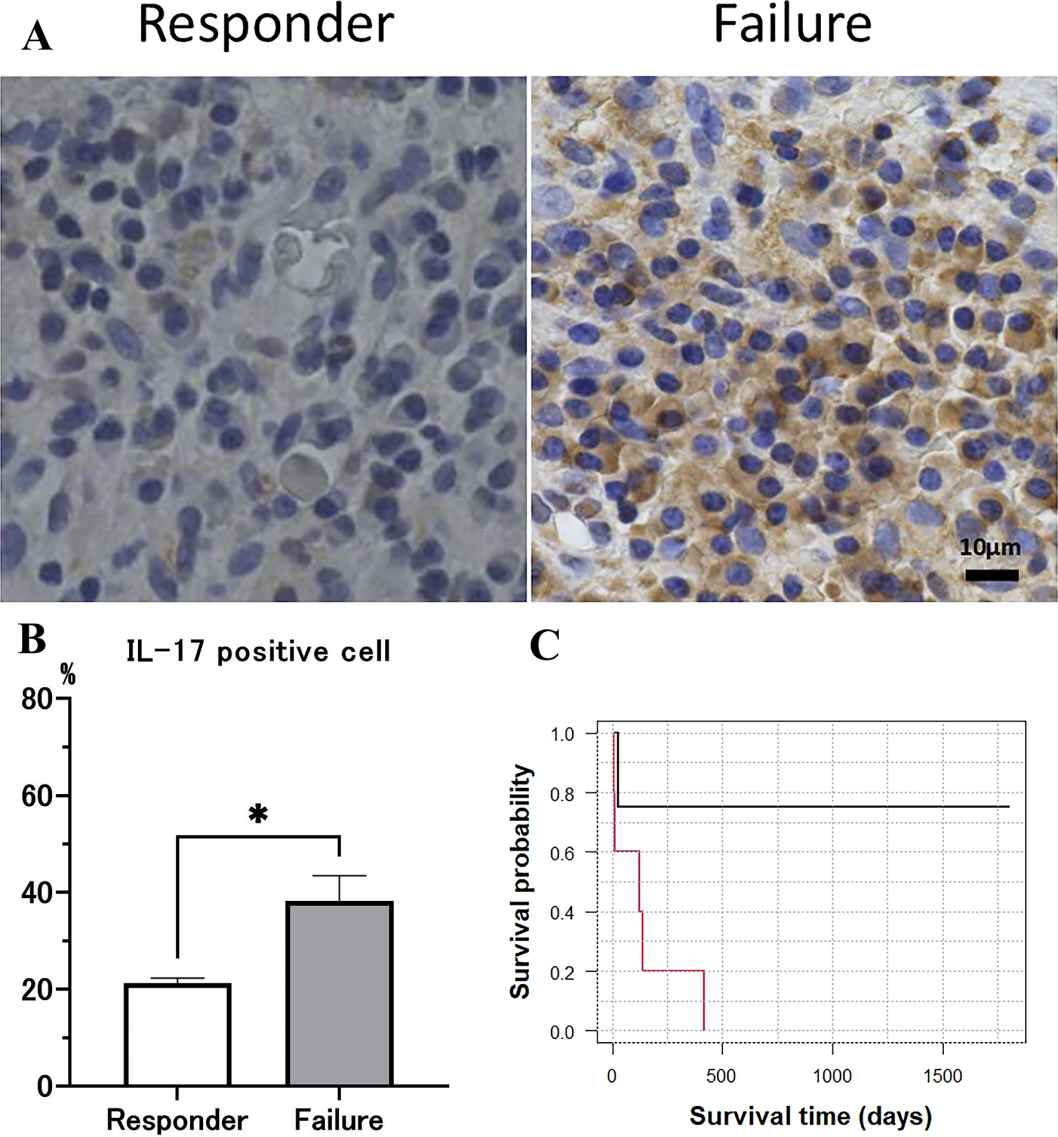
(A) Representative immunostaining for IL-17A in biopsy specimens obtained from the colorectal mucosa before treatment. IL-17A positivity was markedly elevated in the failure group compared to the responder group. (B) Proportion of IL-17A-positive mononuclear cells. The failure group exhibited a significantly higher proportion than the responder group (38.2% vs. 21.2%). (C) Probability of tofacitinib (TOF) continuation stratified by IL-17A positivity. Patients with ≥30% IL-17A-positive cells had a higher probability of continuation compared to those with <30%.

A previous study presented at European Crohn’s and Colitis Organization (ECCO) congress in 2022 suggested that high levels of IL17A-expressing T cells before treatment was associated with failure to achieve response upon TOF treatment^9^. To examine the impact of IL-17 A-positive cells in response to TOF, the number of IL-17 positive mononuclear cells in biopsy specimens obtained from the colorectal mucosa before treatment were counted (Fig. 2A). First, it was verified whether there were any differences in patient backgrounds between the responder and failure groups (Table 2). No significant differences were observed in age, sex, disease duration, disease location, anti-TNF history, concomitant medication, clinical score, or laboratory investigation results between the two groups. Next, we examined whether the degree of IL-17 A-expressing immune cells in the colorectal mucosa was different. The proportion of IL-17 A positive mononuclear cells was higher in the failure group than among responders (38.2% vs. 21.2%) (Fig. 2B C, Table 2, Supplementary Table 2). In addition, the proportion of patients who exhibited ≥ 30% IL-17 A-positive cells in the colorectal mucosa was greater than those who had < 30% (83.3% vs. 0%) (Table 2). ROC curve analysis was performed to assess the value of the proportion of IL-17 A-positive mononuclear cells in predicting the response to TOF. Results demonstrated an area under the ROC curve (AUC) of 0.8333, sensitivity of 100%, specificity of 83.3%, and cut-off value of 29.0 (data not indicated).

### In vitro analysis from RNA-sequence public data regarding the influence of TOF on peripheral T cells　

**Fig. 3:**
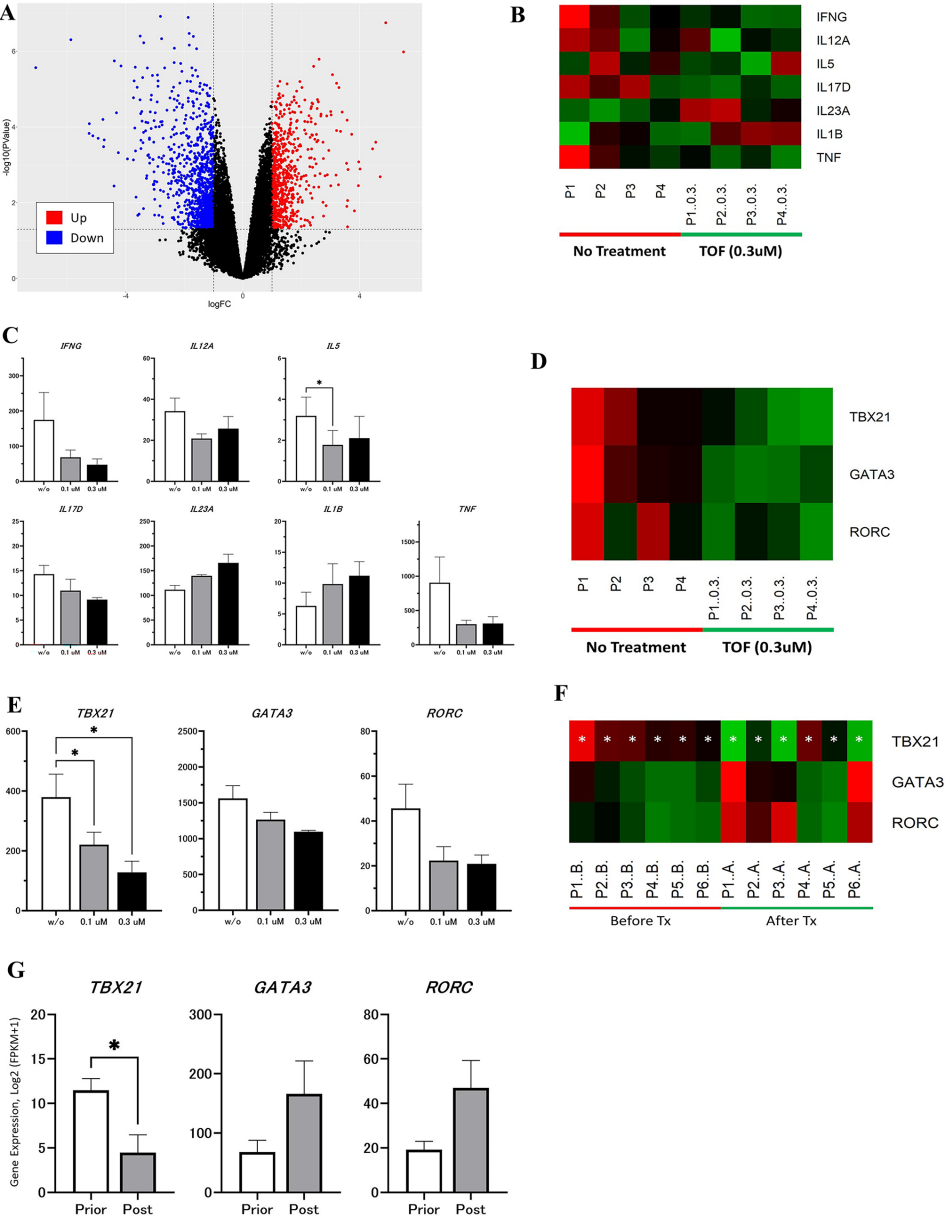
(A–E) Gene expression profiles from GSE60482 dataset, in which naïve CD4⁺CD45RA⁺CD45RO⁻ T cells are isolated and sorted from whole blood of healthy donors and cultured under specific conditions with or without tofacitinib (TOF) treatment. (A) Volcano plots of differentially expressed genes (DEGs) in the GSE60482 dataset. (B) Heatmap shows the effects of TOF on cytokine gene expression in the cultured immune cells. (C) Differences in cytokine gene expression between immune cells cultured with and without TOF treatment. (D) Heatmap shows the effects of TOF on transcription factor gene expression in the cultured immune cells. (E) Differences in transcription factor gene expression between immune cells cultured with and without TOF treatment. (F–G) Gene expression profiles from the GSE169146 dataset, in which RNA sequencing are performed on skin biopsies from six patients treated with TOF monotherapy (5 mg BID) for six months. (F) Heatmap shows the effects of TOF on transcription factor gene expression in skin biopsy samples. (G) Differences in the transcription factor gene expression levels between skin biopsies with and without TOF.

The GEO database was searched for RNA-seq profiles; more specifically, the gene expression profile from GSE60482, in which naïve CD4 + CD45RA + CD45RO- T cells were isolated and sorted using a flow cytometer from whole blood of healthy donors and cultured under specific conditions with and without TOF treatment. After obtaining the gene expression profile, DEG analysis was performed. A total of 933 DEGs were identified after analyzing the GEO dataset (GSE60482), and a large number of genes (*n* = 513) were downregulated after treatment with TOF (Fig. 3A). Subsequently, the effects of TOF on cytokine expression in immune cells was analyzed. Following treatment of immune cells with TOF at concentrations of 0.1 and 0.3 µM, while statistical significance was not achieved, a dose-dependent downregulation of IFN-gamma, a Th1-derived cytokine, was observed (Fig. 3B C). The expression of transcription factors involved in T-cell differentiation, such as *TBX21*, *GATA3*, *RORC* was then analyzed. Among these 3 genes, only *TBX21* (a key transcriptional activator of Th-1 cell differentiation) was significantly downregulated after treatment with TOF (Fig. 3D and E). In the pathway analysis, Type II interferon signaling (WP619) was affected, whereas no significant changes were observed in signaling pathways associated with Th17 (Supplementary Table 3). These findings indicated that TOF exerts a more pronounced influence on Th1 cells compared with IL-17-producing Th17 cells, potentially resulting in loss of control over excessive Th17-related disturbance.

### Public data on long-term influence of TOF treatment

To investigate the influence of TOF on patients, the gene expression profile was obtained from the GSE169146 dataset, in which skin biopsies from six patients who were treated with TOF monotherapy (5 mg BID) for 6 months were used for RNA-seq analysis. Consistent with in vitro examination, only *TBX21* gene expression was significantly downregulated after 6 months of treatment with TOF (Fig. 3F and G), whereas *RORC* (key transcriptional activator of Th-17 cell differentiation) gene expression was not.

## Discussion

The management of UC is complex, with various therapeutic options, such as corticosteroids, immunomodulators, biologics, and JAK inhibitors. This complexity stems from the inherent difficulty in predicting therapeutic responsiveness before initiating treatment. Our study focused on delineating factors that influence a favorable response to TOF in patients with UC. Immunohistological examination revealed that the presence of IL-17 A-positive cells served as a predictive indicator of early failure of TOF during treatment for UC.

The current literature supports the concept that Th17 cells play a pivotal role in the pathogenesis of inflammatory bowel disease (IBD). The presence of Th17 cells in the intestinal lamina propria, along with the constitutive production of IL-17 A in mice, has been documented^10^. In the context of pathogenesis, a detailed analysis of murine models of IBD revealed elevated levels of IL-23^11^ and IL-17 A^12^. Notably, a recent study provided insights into the contribution of IL-23-dependent Th17 responses to the pathogenesis of colitis, especially in the later phase^13^. Consistent with these animal studies, a human study using tissue samples demonstrated upregulated levels of IL-17 A in patients with UC^14^. Mucosal expression of mRNA IL-17 A was 99.8 times higher in patients compared with controls, while the mRNA expression of IFN-gamma and IL-13 increased by factors of merely 12.4 and 6.7, respectively^15^. Moreover, serum IL-17 A levels in treatment-naïve patients with UC not only reflect disease severity at disease onset but also predict the disease course over the ensuing 3 years^15^.

JAK inhibitors, such as TOF, are increasingly being used to treat UC. The selectivity of the regulated pathways among JAK inhibitors may affect treatment outcomes. Detailed investigations using human cells have revealed that TOF inhibits JAK1, JAK2, JAK3 and, to a lesser extent, TYK2, whereas in vivo studies emphasize its preferential inhibition of the JAK1 and JAK3 functions^16^. Disruption of signals linked to JAK3- and JAK1-dependent cytokines include IL-2, IL-6, IFNs, IL-12, IL-4, IL-7, IL-15, and IL-21. Comprehensive pharmacological analysis also underscores that JAK inhibitors most potently inhibit the JAK1/TYK2-dependent pathway, with TOF emerging as the most potent inhibitor of JAK1/3-dependent cytokines among several JAK inhibitors^17^. However, studies investigating the immunosuppressive effects of TOF on JAK2/TYK2 dimer are limited. The IL-23 receptor complex, intricately associated with the JAK2/TYK2 pathway, predominantly facilitates STAT3 phosphorylation and, to a lesser extent, STAT1, STAT4, and STAT5 phosphorylation^18^. The pathological consequences of excessive IL-23 signalling are associated with its capacity to stimulate the production of inflammatory mediators linked to Th17 cells. These mediators include IL-17, IL-22, granulocyte-macrophage colony-stimulating factor (GM-CSF), and TNF-alpha among target populations, predominantly Th17 or IL-17-secreting TCRgd cells (Tgd17)^19^. This foundational verification suggests that TOF may not be able to exert optimal control over excessive Th17 cell responses stemming from aberrant IL-23 activation. In accordance with this concept, patients with an abundance of IL-17 A-positive mononuclear cell infiltration exhibited a lack of positive response to TOF treatment in our investigation. Moreover, our results are supported by a previous study using a single-cell RNA sequence, in which high levels of IL17A-expressing T cells at baseline were significantly correlated with the failure to achieve a response to TOF treatment^9^. In this study, six non-responders were identified, among whom one patient was administered a biological agent with inhibitory effects on IL-23 (ustekinumab), subsequently following TOF treatment. In this case, ustekinumab led to a marked improvement in symptoms during the induction and maintenance phases.

Given that advanced medications, including biologics and JAK inhibitors, are not universally effective, are associated with rare but serious side effects, and incur high costs, it is crucial to selectively administer treatments to patients with the highest likelihood of a favorable response. These considerations underscore the substantial demand for a reliable scale to predict the treatment response. Current indicators of a positive response include age, sex, body weight, smoking habits, disease duration, disease location, disease severity, and extraintestinal manifestations^20^. In addition to these physical factors, multiple studies have assessed the mucosal expression profiles in patients with UC to predict treatment responses. Gene-array analysis using pre-infliximab treatment of rectal mucosal biopsy samples from patients with active UC identified a panel of the top 5 DEGs that can indicate the responsiveness: osteoprotegerin (TNFRSF11B), stanniocalcin-1 (STC1), prostaglandin-endoperoxide synthase2 (COX2), IL13Ra2, and IL11. This analysis demonstrated the capability to distinguish responders from non-responders with 95% sensitivity and 85% specificity^21^. In another study, involving 67 patients with UC undergoing anti-TNF treatment, mucosal healing was associated with lower mucosal expression of TBX21 (a Th1-related transcription factor) and higher expression of RORC (a Th17-related transcription factor) before treatment^22^. However, it has been also shown that high mRNA expression of both mucosal IFN-gamma and IL-17 A in biopsies obtained before treatment was linked to the response to anti-TNF induction therapy in patients with UC^23^. While our study demonstrated that the activation of the Th17 cell lineage contributes to resistance to TOF treatment, predictors of anti-TNF treatment may indicate the opposite.

Finally, recent studies have underscored the potential of histological examinations in predicting treatment responses among patients with UC. Gaujoux et al. reported that the altered abundance of plasma cells and inflammatory macrophages in the intestinal mucosa distinguished responders from non-responders to anti-TNF^24^. Tew et al. demonstrated that the mucosal expression of integrin E (ITGAE), examined by immunohistochemistry, can predict the treatment response to etrolizumab (a humanized monoclonal antibody that selectively binds the b7 subunit of both heterodimeric integrins a4b7 and aEb7)^25^. These findings suggest the potential existence of more refined histological predictors, such as the abundance of IL-17-positive mononuclear cells, to identify individuals who would well respond to treatment.

Our study had several limitations, the first of which was its small sample size, in addition to its retrospective, single-center design.

In conclusion, results of this study present evidence supporting the clinical applicability of an abundance of IL-17 A-positive mononuclear cells in the colonic mucosa to predict responsiveness to TOF treatment. We hope that further studies will confirm our findings and our study contributes to the development of new methods for precise identification of suitable candidates for TOF treatment.

The gene expression data used in this study are publicly accessible in the GEO database under accession codes GSE60482 and GSE169146. The data can be retrieved at https://www.ncbi.nlm.nih.gov/geo/query/acc.cgi?acc=GSE60482 and https://www.ncbi.nlm.nih.gov/geo/query/acc.cgi?acc=GSE169146.

**Table 1:** Demographic data (*n* = 9).

	Mean (SD) or *n* (%)
Age, y	37.4 (16.4)
Female	3 (33.3)
BMI, kg/m^2^	20.5 (3.3)
Duration of disease, m	91.4 (107.8)
Current smoker	0 (0)
Disease location	
Total colitis	7 (77.8)
Left-sided colitis	2 (22.2)
Proctitis	0
Previous exposure to advanced therapies (number)	1.3 (0.5)
Previous exposure to advanced therapies (agents)	
Anti-TNF	6 (66.7)
Tacrolimus	2 (22.2)
Anti-integrin	2 (22.2)
IL-12/IL-23 antagonist	1 (11.1)
JAK inhibitor	0 (0)
Concomitant medications	
5-ASA	8 (88.9)
Corticosteroids	1 (11.1)
Clinical score	
Partial Mayo score	5.8 (2.3)
Endoscopic Mayo score	2.7 (0.5)
Laboratory test	
C-reactive protein, mg/dl	2.1 (3.0)
Albumin, g/dl	3.6 (0.8)
Hemoglobin, g/dl	12.3 (1.8)

**Table 2:** Clinical factors contributing to tofacitinib response.

	Responder (*n* = 3)	Failure (*n* = 6)	*p* -value
Age, y	48.7 (12.5)	31.8 (11.5)	N.S.
Female	2 (66.7)	1 (16.7)	N.S.
BMI, kg/m^2^	19.6 (0.7)	21.0 (4.0)	
Histological evaluation			
Percentage of IL-17 A (+) cell	21.2 (1.9)	38.2 (12.7)	< 0.05
Less than 30% of IL-17 A (+) cells	3 (100)	1 (16.7)	< 0.05
Duration of disease, m	52.7 (10.1)	110.8 (131.2)	N.S.
Disease location			
Total colitis	2 (66.7)	5 (83.3)	N.S.
Left-sided colitis	1 (33.3)	1 (16.7)	
Previous exposure to ATx (number)	1.0 (0.0)	1.5 (0.5)	N.S.
Previous exposure to ATx (agents)			
Anti-TNF	3 (100)	3 (50.0)	N.S.
Tacrolimus	0 (0)	2 (22.2)	N.S.
Anti-integrin	0 (0)	2 (22.2)	N.S.
IL-12/IL-23 antagonist	0 (0)	1 (11.1)	N.S.
JAK inhibitor	0 (0)	0 (0)	N.S.
Concomitant medications			
5-ASA	3 (100)	5 (83.3)	N.S.
Corticosteroids	1 (33.3)	0 (0)	N.S.
Clinical score			
Partial Mayo score	5.3 (2.1)	6.0 (2.5)	N.S.
Endoscopic Mayo score	2.7 (0.6)	2.7 (0.5)	N.S.
Laboratory test			
C-reactive protein, mg/dl	1.0 (0.9)	2.7 (3.5)	N.S.
Albumin, g/dl	3.1 (0.8)	3.8 (0.8)	N.S.
Hemoglobin, g/dl	11.5 (1.0)	12.7 (2.1)	N.S.

## Electronic supplementary material

Below is the link to the electronic supplementary material.

Supplementary Material 1

Supplementary Material 2

Supplementary Material 3

## Data Availability

The gene expression data used in this study are publicly accessible in the GEO database under accession codes GSE60482 and GSE169146. The data can be retrieved at https://www.ncbi.nlm.nih.gov/geo/query/acc.cgi? acc=GSE60482 and https://www.ncbi.nlm.nih.gov/geo/query/acc.cgi? acc=GSE169146.
