# Supplementary material for: Activated type 17 helper T cells affect tofacitinib treatment outcomes
Source: Sci Rep. 2025 Feb 19;15:6112. doi: 10.1038/s41598-025-87076-7 (PMC11840122; doi:10.1038/s41598-025-87076-7)
Supplement: Supplementary file 1 — Supplementary Material 1 [file 41598_2025_87076_MOESM1_ESM.docx]

Supplementary Table 1

|  |  | The start date of TOF treatment | The end date of TOF treatment | The duration of TOF treatment (days) |
| --- | --- | --- | --- | --- |
| #1 | Failure | 2018/9/20 | 2019/2/4 | 137 |
| #2 | Failure | 2019/7/18 | 2019/8/13 | 26 |
| #3 | Failure | 2019/9/25 | 2019/10/4 | 9 |
| #4 | Failure | 2019/12/26 | 2020/4/26 | 122 |
| #5 | Failure | 2020/9/25 | 2020/10/8 | 13 |
| #6 | Failure | 2021/2/12 | 2022/4/7 | 419 |
| #7 | Responder | 2018/10/25 | - | Ongoing |
| #8 | Responder | 2019/10/29 | - | Ongoing |
| #9 | Responder | 2019/11/21 | - | Ongoing |
| #10 | Responder | 2018/11/9 | - | Ongoing |
| #11 | Responder | 2019/9/26 | - | Ongoing |
| #12 | Responder | 2020/7/16 | - | Ongoing |
