# Supplementary material for: Activated type 17 helper T cells affect tofacitinib treatment outcomes
Source: Sci Rep. 2025 Feb 19;15:6112. doi: 10.1038/s41598-025-87076-7 (PMC11840122; doi:10.1038/s41598-025-87076-7)
Supplement: Supplementary file 2 — Supplementary Material 2 [file 41598_2025_87076_MOESM2_ESM.docx]

Supplementary Table 2

|  |  | The proportion of IL-17A positive cells (％） | | | |
| --- | --- | --- | --- | --- | --- |
|  |  | field1 | field2 | field3 | Average |
| #1 | Failure | 51.96078431 | 52.38095238 | 19.71830986 | 41.35334885 |
| #2 | Failure | 16.79389313 | 17.64705882 | 20.93023256 | 18.4570615 |
| #3 | Failure | 73.45679012 | 47.5 | 52.35294118 | 57.76991043 |
| #4 | Failure | 40.0 | 36.69064748 | 29.46428571 | 35.38497773 |
| #5 | Failure | 33.84615385 | 47.27272727 | 42.10526316 | 41.07471476 |
| #6 | Failure | 18.86792453 | 46.32352941 | 40.83333333 | 35.34159576 |
| #7 | Responder | 12.5 | 27.82608696 | 27.88461538 | 22.73690078 |
| #8 | Responder | 11.0 | 14.16666667 | 32.17391304 | 19.11352657 |
| #9 | Responder | 28.67647059 | 16.23376623 | 20.66115702 | 21.85713128 |
