# Supplementary material for: Activated type 17 helper T cells affect tofacitinib treatment outcomes
Source: Sci Rep. 2025 Feb 19;15:6112. doi: 10.1038/s41598-025-87076-7 (PMC11840122; doi:10.1038/s41598-025-87076-7)
Supplement: Supplementary file 3 — Supplementary Material 3 [file 41598_2025_87076_MOESM3_ESM.docx]

Supplementary Table 3

| ID | Description | *p* value | Gene Symbol | Count |
| --- | --- | --- | --- | --- |
| WP5193 | Cholesterol synthesis disorders | 1.78E-07 | EBP/IDI1/MSMO1/HMGCS1/DHCR7/LBR/DHCR24/CYP51A1/HMGCR/MVK/PMVK/ FDPS | 12 |
| WP4718 | Cholesterol metabolism with Bloch and Kandutsch-Russell pathways | 1.00E-06 | EBP/FADS1/SCD/IDI1/MSMO1/FADS2/ACSL3/HMGCS1/ABCA1/ACSL4/DHCR7/LBR/ABCG1/DHCR24/CYP51A1/HMGCR/ MVK/PMVK/FDPS | 19 |
| WP5333 | Enterocyte cholesterol metabolism | 1.76E-05 | EBP/IDI1/LDLR/MSMO1/HMGCS1/ABCA1/DHCR7/LBR/DHCR24/CYP51A1/HMGCR/ MVK/PMVK/FDPS | 14 |
| WP197 | Cholesterol biosynthesis pathway | 4.72E-05 | IDI1/MSMO1/HMGCS1/DHCR7/CYP51A1/HMGCR/MVK/PMVK/FDPS | 9 |
| WP5413 | IL-24 Signaling pathway | 0.000276861 | GZMB/ATM/COL6A2/CYCS/CDK6/S100A6/MST1/CD44/ITGAM/IRF4/DSTN/EIF2AK2/VIM/BCL2L11/MYC/TXN/CD83/HSPE1/EIF4E/PMAIP1/AP3S1/XBP1/CHEK1/PRDM1/PRF1/EIF2S1/CFLAR/ABCB1/CD200/CDC6/SOCS3/CASP3/HSP90AA1/EEF1E1/COX7B/ATG5/BAX/ATF4/BCCIP/EIF4EBP1/CD82/PHB1/PARP1/FBP1/CDC25A/PECAM1/FEN1/RB1/MCM10/AK1/PCNA/TIMP1/ODC1/STAT1 | 54 |
| WP4190 | Mevalonate arm of cholesterol biosynthesis pathway | 0.000444836 | IDI1/ACAT1/HMGCS1/HMGCR/MVK/PMVK/FDPS | 7 |
| WP619 | Type II interferon signaling | 0.000602619 | JAK2/IRF4/EIF2AK2/CXCL9/IFI6/PTPN11/ PSMB9/GBP1/SOCS3/IFIT2/OAS1/STAT1 | 12 |
